# The networked researcher, the editorial manager, and the traveller: the profiles of international political scientists and the determinants of internationalisation

**DOI:** 10.1057/s41304-022-00368-8

**Published:** 2022-03-03

**Authors:** Filippo Tronconi, Isabelle Engeli

**Affiliations:** 1 Department of Social and Political Sciences, University of Bologna, Bologna, Italy; 2 Department of Politics, University of Exeter, Exeter, UK

**Keywords:** Europeanisation, Globalisation, Higher education, Political science as a profession

## Abstract

The concept of internationalisation, when referring to the work of social scientists within academic institutions, takes on different meanings and involves different activities. This contribution aims to shed light on the international activities of political scientists across Europe and to investigate the various meanings and practices of internationalisation. The analysis relies on the PROSEPS survey, involving some 1,800 political scientists across 37 European countries. We identify three distinct profiles of international scholars: the networked researcher, the editorial manager, and the traveller. These profiles differ according to 1) the building of international research networks, 2) the involvement in the activities of the international publishing industry, 3) the research and teaching exchanges with foreign academic institutions. Determinants, such as gender, family status, career stage, availability of institutional and financial support, and geographical location, are considered as potential drivers or inhibitors of internationalisation. Our analysis shows that the internationalisation of academic practices follows contrasting paths according to the type of international profile.

## Introduction: the quest for internationalisation in political science

The idea that science is an intellectual enterprise overcoming national borders is something that we often take for granted, without requiring any further justification or specification. Although the international mobility of students and scholars is as old as universities themselves (De Ridder-Symoens 1991), the international academic flows have significantly increased after the end of World War Two (Lanzendorf and Kehm 2010; Maringe and Foskett 2010; Norris 2020a). Academic international exchanges have been particularly intensified in Europe, thanks to the process of European integration. Since the 1970s, the European Commission has been keen to promote faculty contacts across member states and the development of joint curricula. The establishment of the well-known Erasmus programme (1987) and the Bologna Declaration (1999) have been historical milestones in the process of harmonisation of national systems of higher education (Rezaev 2010).

Of course, internationalisation does not necessarily overlap with physical cross-border mobility. Publishing with international co-authors, as well as with international journals and publishing houses, is a key indicator of internationalisation. And so is being funded from a supranational organisation and being involved in a teaching programme in a language that is different from the majority language of one’s own country. Internationalisation has never been more distinct from travelling. The digital revolution has provided students and scholars with an ever-growing range of tools for connecting and cooperating with increasing ease (Norris 2020a). The COVID-19 pandemic has certainly accelerated such forms of cooperation in addition to putting to the forefront virtual conferences and events.

The social sciences are no exception to this broader trend (Kuhn and Weidemann 2015). Indeed, transnational collaboration is a way to overcome the perils of ethnocentrism and western-centrism (Smelser 2003). Internationalisation allows a healthy circulation of ideas, an exchange of views and experiences that can only represent enrichment from the personal and intellectual point of view. Being exposed to different traditions, academic institutions, social, political, and economic realities means putting in perspective our own personal experiences and beliefs. This, ultimately, allows a better understanding of the world that is the fundamental mission of political science, a discipline that is comparative by its nature.

While the merits of internationalisation could be seen as self-explanatory in the context of the knowledge society, it is nevertheless worth noting that academic international cooperation is sometimes considered as a compounding factor of existing inequalities (Altbach and Knight 2007; Bilecen and Mol 2017; Norris 2020a). Brain drain, the one-way flow of highly skilled persons from developing to developed countries, is the most visible downside of internationalisation, and a frequent concern within the European Union and elsewhere (Ienciu and Ienciu 2015). In a recent study of global political science, Norris (2020a, 137–139) emphasises the growing category of “academic migrants”, i.e. the academics who work in a different country from the one they were born in or even study in. Norris (2020a, 138) highlights the fact that academic “migration” has largely remained a one-way process towards Northern and Western Europe. Our study confirms this finding, as we will see. Segmentation of scholarly communities within countries is another potentially negative consequence of mobility. By segmentation of scholars, we mean the establishment of two separate groups: a first group of scholars who are highly internationalised in terms of research and publications, with frequent opportunities to physically cross the borders of their own country to meet colleagues abroad in conferences and workshops; and a second group who, instead, rarely or never move abroad, do not participate in international networks, and who are not particularly aware of global trends of the discipline. In short, there are instances when internationalisation might lead to increased imbalances between and within countries and academic institutions (see Boncourt et al. in this symposium on the case of France).

This article contributes to this debate by examining the relevance of the international dimension of scholarly work in contemporary European political science. Carried out in 2018, the PROSEPS survey offers an opportunity to map how European political scientists perceive their international academic environment (see also Norris 2020a), its relevance for the advancement of knowledge and, more prosaically, for its enablement of career advancement. Our analysis reveals three profiles of international scholars: the networked researcher, the editorial manager, and the traveller. These profiles differ according to 1) the building of international research networks, 2) the involvement in the activities of the international publishing industry, and 3) the research and teaching exchanges with foreign academic institutions. We also explore a number of explanatory factors that enhance or hamper opportunities for international collaboration. While the empirical tools adopted focus our attention at the individual level, we will see that academic institutions have powerful tools to increase opportunities for international mobility and cooperation of its affiliates. Indeed, one of the main findings of this work is that enhancing institutional support to international activities is an effective driver of internationalisation, just as Norris’ study pointed out for the global level (2020a).

The article is organised as follows: we first disentangle the different meanings associated with the concept of internationalisation of scholarly work. After a short description of the survey on which the empirical analyses are carried out, we then identify three different profiles of international political scientists. Finally, we turn our attention to the determinants of internationalisation, showing that they are at least partially different for each of the three profiles.

## What we talk about when we talk about academic internationalisation

This article aims at mapping the extent to which European political scientists are internationalised and it investigates the main drivers of internationalisation. The term internationalisation, though, carries a range of meanings and has been used in contrasting ways. In her (non-exhaustive) list, Jane Knight (2004: 6) mentions prominently: the academic activities that involve transnational mobility for students and/or teachers; the international linkages, partnerships, and projects—both in teaching and research programmes; the establishment of university branches and campuses outside the original country; as well as the inclusion of an international, intercultural or global dimension into the curriculum and teaching/learning processes. To these, we add activities that are specifically related to academic research: the effort to capture international/supranational funding; the establishment and lead of international research groups; publishing in non-national journals with authors from different countries; and participation in non-national conferences.

The quest for a conceptual clarification is not just the result of the usual academic pedantry. The point is that each of these internationalising practices (and others that could be added) might appeal to different types of scholars and might require a different set of skills and resources. It is thus necessary to disentangle the meanings of internationalisation. To do so, we propose to draw on insights of existing practices rather than on a priori deliberation. The richness of the data of the PROSEPS survey allows us to investigate current practices of political scientists across Europe. Starting from self-reported scholarly activities, we look for different patterns of internationalisation and offer a parsimonious description of the actual meaning of internationalisation on the ground across European political science.

## The PROSEPS survey

The PROSEPS (PROfessionalization and Social impact of European Political Science) project aims at building a network of scholars—mostly political scientists and political sociologists—to study the internationalisation and social impact of political science in Europe. It focuses on 1) the transformation of the academic subject, 2) the social and media visibility of its research outputs, 3) the international mobility and circulation of its researchers, 4) the applicability and concrete applications of the work of political scientists. Funded as a COST Action, the project ran between 2016 and 2020 and involved scholars from 40 European countries.^1^ One of its most ambitious research goals was the production of a large-scale survey among European political scientists addressing their vision and perception of the discipline and of its social status. In addition to questions related to educational background, career trajectory, and research interests, batteries of questions were asked about internationalising practices, participation in public debate, and consultancy activities. The survey was carried out between May and December 2018, with the CAWI method. 11,827 European political scientists were contacted, of which 2,216 returned a complete response (20.7% response rate).^2^

As far as internationalisation is concerned, the PROSEPS survey includes a large number of useful indicators (17), covering a broad range of internationalising activities related to teaching, research, and leadership. Table 1 provides a list of the indicators that have been used in our analyses. For each one, respondents were asked how many times the activity had been carried out during the previous three years. After dropping the cases of respondents who did not hold a PhD at the time the survey was administered (mostly doctoral students, or, in some countries, adjunct or emeritus professors) and some other cases with missing information, we are left with 1,868 respondents from 37 European countries, ranging from four respondents from Montenegro to 207 from the UK. While the response rate is satisfactory and in line with usual response rates for this kind of survey (and this is the first survey of its kind to cover entire Europe), we are still analysing responses from a minority of political scientists. Self-selection biases most probably matter. In addition, self-reporting may tend to amplify particular responses (social desirability bias). After all, political scientists have been extensively trained in writing performance reports for annual reviews, highlighting self-promoting activities. With these caveats in mind, our analyses are to be considered as illustrative of the current trend in internationalising practices rather than an iron law. This said, we believe our analyses shed light on the various practices regarding internationalisation and show that there is more than one road leading to Rome. Our analyses also further contribute to attracting attention to the inequalities across Europe and the work that remains to be accomplished for fully achieving an inclusive European Political Science (Boncourt et al. 2020; Norris 2020a).

**Table 1:** Indicators of internationalisation. Principal component analysis. *Source*: Authors’ elaboration on PROSEPS survey

Indicators	Rotated components		
	1	2	3
	Eigenvalue 5.03	Eigenvalue 1.45	Eigenvalue 1.29
1. Published with international co-authors	0.633		
2. Published in English	0.774		
3. Published in a language (not English) other than the principal language of your academic system			
4. Participated (presented a paper or acted as discussant) in an international conference	0.616		
5. Gone on a research stay abroad of at least 2 weeks			0.803
6. Taught outside the country where you work			0.669
7. Articles in peer-reviewed international journals	0.768		
8. Chapters in edited books published by international publishing houses	0.510	0.471	
9. Monographs published by international publishing houses		0.601	
10. Partner or subcontractor of a research project funded by international institutions (H2020, ERC, COST, etc.)			
11. Member of an international research network within your field of interest	0.524		
12. Reviewer of project applications funded by international or other country's institutions		0.497	
13. Referee for an international peer-reviewed journal	0.672		
14. Editor for an international peer-reviewed journal		0.456	
15. Reviewer for an international publishing house (in a country different from where you currently reside)		0.570	
16. Book series editor for an international publishing house (in a country different from where you currently reside		0.626	
17. Time spent working (performing research or teaching duties) in countries other than the one in which you reside			0.811

Rotated Component Loadings (varimax method)

Cronbach’s alpha: Component1 = 0.81; Component2 = 0.63; Component3 = 0.73

## The three types of international scholars: the networked researcher, the editorial manager, and the traveller

We relied on a principal component analysis (PCA) with varimax rotation to identify the main types of international scholars across European political science. PCA is a statistical technique that is widely used to reduce dimensionality in cases of rich and complex information. In our analysis, PCA is useful for disentangling different dimensions of a concept while keeping as much information that is contained in the original set of the 17 indicators of internationalisation as possible. In other words, the goal of PCA is to maximise parsimony while preserving most of the richness of the original information. When several factors are considered, PCA allows to organise the maximum number of factors into the minimum number of underlying dimension(s). The first component retains the maximum possible information, the second component retains the maximum of the remaining information, and so forth.

Table 1 displays the results of our principal component analysis based on 17 indicators of internationalisation. Three components have an Eigenvalue larger than one and explain 45.7% of the variance. Simply put, the Eigenvalues are coefficients that provide an indication about the amount of variance carried in each principal component. The higher the Eigenvalue is, the higher the significance of the principal component is, and the larger amount of variance is carried by the component. The first component will always have the higher significance. Table 1 reports the components whose loading is above 0.40, a standard cut-off point. Two indicators (*Published in a language (not English) other than the principal language of your academic system* and *Partner or subcontractor of a research project funded by international institutions*) do not load sufficiently on either of the three components. It means that organising them together with other factors does not bring enough to any of the three main components. For this reason, they will not be considered in the following analyses.

What does Table 1 suggest from a substantive point of view? The analysis reveals three different types of internationalised scholars, each of them displaying key distinguishing features regarding internationalising practices. We label the first type of scholar the *international networked researcher*. Internationalised scholars belonging to this first type tend to publish in peer-reviewed international journals, and often with international co-authors (first column in Table 1). Moreover, these scholars also serve as referees for international journals. They do not disdain publishing chapters in international edited books and are frequent participants in international conferences.

The *international editorial manager* is our second type of internationalised scholar (second column in Table 1). This group of scholars serves as editors of international journals and book series and as reviewers for publishing houses. The international editorial managers prefer to publish their own work in the form of monographs (as opposed to journal articles). Our third type of internationalised scholar is characterised by two specific cross-border activities, namely travelling for long periods of time as a visiting researcher in foreign institutions and teaching or having taught abroad. We call this third type the *international traveller*.

How can we explain these differences in internationalising practices? The next section investigates how the three types of internationalised scholars are geographically distributed, as well as which individual and institutional resources are correlated with each type. Beyond geography, we zoom in on three main factors that are often suggested to have an impact on the type and degree of internationalisation: family responsibilities (with potential differentiated effects for men and women), institutional support from the university of belonging, and career stage.

## Geography and beyond: what explains contrasting practices in internationalisation?

**Fig. 1:**
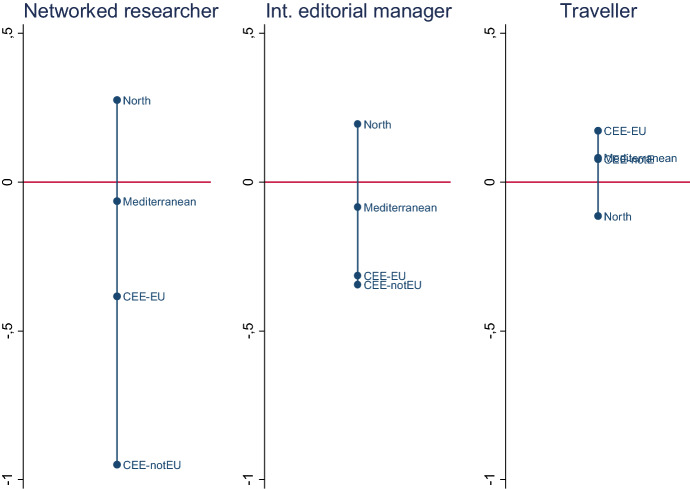
The three types of international political scientists, by geographical area. *Note*: standardised values (1 means one standard deviation above or below the average). *Source*: Authors’ elaboration on PROSEPS survey

For the purpose of this investigation, we identify three broad European regions: Northern Europe, the Mediterranean countries, and Central and Eastern Europe (CEE). In addition, we divide CEE into two sub-groups according to European Union membership.^3^ Figure 1 pictures the distribution of each type of international scholar across the four categories. The dark line represents the overall average. An area located above the dark line indicates that a specific type of international scholar is strongly present in this area. Figure 1 provides us with a precious insight on the regional variations in internationalising practices at play. Northern European countries have the most networked researchers and international editorial managers, while scholars based in non-EU Eastern countries are located at the bottom of both rankings. Being involved in international research networks (which includes publishing in international journals) is particularly challenging for scholars working in this region, while the differences are less remarkable for the other two types of international political scientists. It is noteworthy, however, how the geographical distribution of travellers differs. Scholars located in the Northern countries are less ready to go to teach and do research abroad for long periods, while this is the most frequent activity for Central and Eastern European scholars belonging to the EU and, to a lesser extent, for scholars working in non-EU eastern states and the Mediterranean countries. This presumably reflects the attractiveness (and probably the availability of financial resources) of many universities in Northern Europe and the prestige associated with visiting such universities, and corroborates what Norris (2020a) has found about long-term mobility in the recent study on global political science.

Family responsibilities are another source of contrasting propensity to build an international scholarly profile. Having children might be a particularly strong negative incentive in this sense. Gender is another key factor that may potentially affect international activities, and even more so when combined with family responsibilities. The PROSEPS survey provides information on both these aspects. Regarding family responsibilities, we have re-arranged the available information into four categories, integrating the family status with the presence of children in the same household. It seems reasonable to imagine four different situations, in order of increasing challenges posed to international mobility and activities. Scholars who are single, divorced, or widowed and without children are the least likely to have family responsibilities (category 0 in Table 2).^4^ Married scholars or scholars living with common law partners without children are potentially less involved in major care responsibilities (category 1). Family responsibilities increase for scholars with children living in the same household, when they are married or anyway live with a common law partner (category 3), and even more so when they are single, divorced, or widowed (category 4). Table 2 presents the family status of PROSEPS respondents, divided by gender. As it can be seen, women and men who responded to the survey do not differ much in their family responsibilities with the exception of the higher proportion of single/divorced/widowed women without children living at home.

**Table 2:** Family responsibilities by gender of respondents. *Source*: Author’s elaboration on PROSEPS survey

	Family responsibilities					
	0	1	2	3	Total	*N*
Women	8.1	23.2	51.8	17.0	100.0	*583*
Men	3.9	25.9	53.2	16.9	100.0	*1092*
Total	*5.4*	*25.0*	*52.7*	*17.0*	100.0	*1675*

Legenda for family responsibilities: 0: Single/divorced/widowed with no children living at home; 1: Married/common law partner with no children living at home; 2: Married/common law partner with children living at home; 3: Single/divorced/widowed with children living at home.

The third factor potentially affecting internationalisation is institutional support. In our analysis, institutional support includes a range of actions that an academic institution can take to empower political scientists in their internationalisation endeavour and support their international research and teaching activities. The PROSEPS survey asks European political scientists to state if they have benefitted from any of the following four actions over the previous three years: research or teaching fellowships for international activities; funding for travel to conferences abroad; financial, administrative, or technical support for funded international projects; language editing support. From these responses, we built an additive scale: institutional support is absent where none of these actions have been taken. It reaches its highest score where all these actions have been taken over the previous three years. As Table 3 shows, institutional support varies considerably across geographical areas. The vast majority of political scientists working in Northern Europe enjoy at least some institutional support for their international activities and almost one out of three benefited from a high or very high level of support (values 3 and 4 in the table). In the Mediterranean countries and in the Central-Eastern members of the European Union, the scholars who benefit from a high or very high levels of institutional support are 20% and 25%, respectively. This percentage drops to 6.6% in CEE countries outside the EU, where 42% of scholars do not receive any support for internationalisation.

**Table 3:** Institutional support by geographical area. *Source*: Authors’ elaboration on PROSEPS survey

	Institutional support						
	0 None	1	2	3	4 Very high	Total	*N*
North	11.1	23.8	34.2	24.6	6.3	100.0	900
Mediterranean	14.8	30.9	34.3	15.0	4.9	100.0	492
CEE-EU	14.1	23.7	36.4	19.8	5.9	100.0	354
CEE-not EU	41.8	27.0	24.6	6.6	0.0	100.0	122
Total	14.7	25.9	34.0	20.0	5.5	100.0	1868

The four levels of institutional support represent the number of support actions enjoyed over the last 3 years among the following: Research or teaching fellowships for int’l activities; Funding for travel to conferences abroad; Financial, administrative or technical support for funded int’l projects; Language editing support

In the following multivariate model, we investigate the effect of the four variables discussed above (regional location, gender, family responsibilities, and institutional support) and add another one, the career stage. This last variable is operationalised as a simple dichotomy that distinguishes between tenured and non-tenured scholars. As discussed above, family responsibilities can have differentiated effects on men’s and women’s career trajectories. To take this into account, we introduce an interaction term between gender and career stage in the modelling.

**Fig. 2:**
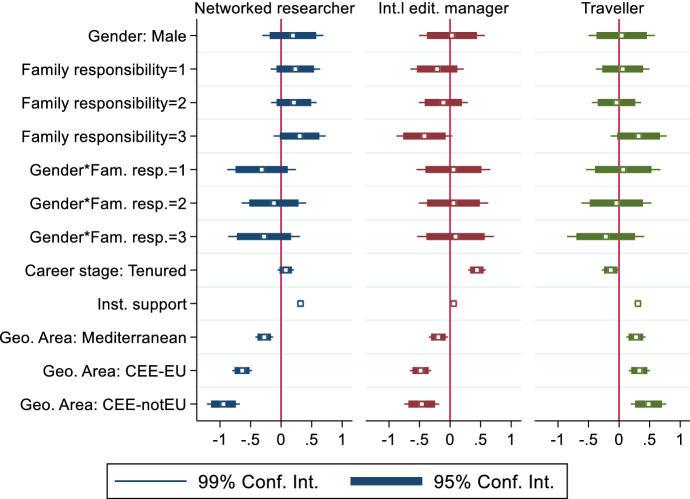
Determinants of internationalisation. Regression models. *Source*: Authors’ elaboration on PROSEPS survey

Figure 2 displays the results of three separate ordinary least square regressions for each of the three profiles of international political scientists that we discussed earlier in this article. The independent variables for each profile are thus equal to the component scores presented in Table 1. The position of each square indicates the standardised coefficient of the respective variable, while the bars and lines indicate the confidence intervals associated with that coefficient. When bars (or lines) do not cross the dark line, it means that they are statistically significant at 95% (or 99%) level.

The first, quite surprising, insight is that neither gender nor family responsibilities seem to have any significant effect on the levels of internationalisation among the PROSEPS respondents. The interaction term is also statistically non-significant, and this for all the three types of international profiles. The only exception is represented by family responsibilities in the case of the international editorial manager. When compared to the absence of family responsibilities (scholars that are single/divorced/widowed and do not have children), the highest level of family burden (single/divorced/widowed with children living at home) has a negative impact on this specific set of activities. It does not exert any effect on the other two sets of activities. This result meets the 95% confidence interval statistical standard. As pointed out earlier, this may also be partially due to self-selection for responding to the survey. Scholars who are juggling between work and family commitments are probably among the least likely to answer a survey about the profession. While care responsibilities are unlikely to be the only driver for self-selection, it is certainly an important one. There are numerous accounts in the literature about how international activities are not always gender- or family-friendly. This said, a recent study from Norris (2021) on the gender gap in research productivity provides precious insights for further situating the meaning of our null result. Norris finds that family and marital status exert no significant effect on the gender gap in the (self-reported) h-index. The study shows that the gender gap in the h-index drastically reduces once career stage and working conditions are taken into account, with no significant gender difference among the youngest cohort.

On the contrary, the variables capturing the career stage, the level of institutional support, and the regional location are all statistically relevant. Tenure is a strong predictor of internationalisation when this refers to editorial management activities. This most probably indicates that experienced scholars are more likely to take on (and to be offered) such responsibilities. International travellers are more commonly found among non-tenured (and often at an earlier career stage) scholars. This is also understandable. Tenured positions often imply teaching duties and institutional responsibilities that cannot be easily bypassed and normally require a physical presence at home university. Institutional support for international activities is also a significant predictor of internationalisation. This time, however, it is more relevant for networked researchers and travellers, and less so for editorial managers. This makes sense, as the four possible types of support included in the PROSEPS survey primarily refer to research activities to be carried out abroad, such as, participation in international conferences, teaching/research fellowships, and support for cooperation in international projects. This finding echoes the argument made by Norris (2021) about the importance of the institutional environment and an individual’s location within it for research productivity. Geography matters as well. In this case, though, it matters in contrasting directions across our three types of international academics. The multivariate models confirm what we have already highlighted in a descriptive form. Remember that geography is operationalised as a dummy variable, thus coefficients represent a comparison between any listed regional area and the North, Northern Europe being selected here as the baseline category. The networked researchers and international editorial managers are found more often in Northern European countries than in other areas. Travellers come, in return, more frequently *from* the three other areas.

## Conclusion

This article aims at advancing our knowledge on the internationalisation of European political scientists. Drawing on the PROSPEPS survey, we have first disentangled three different and complementary set of practices regarding internationalisation and identified three ideal typical profiles: the networked researcher, the international editorial manager, and the traveller. Our investigation about the determinants of the propensity of European scholars to resemble one of the mentioned profiles reveals that some factors (career stage and geography, and to a lesser extent institutional support) exert differentiated directions for each type of international political scientist. While the PROSEPS survey covers the entire area of Europe and the response rate is satisfactory, we nevertheless have to keep in mind potential effects linked to self-selection and self-reporting. Our findings are thus best considered as illustrations of trends in practices rather than an iron law about any successful internationalisation of political science.

Contrary to our expectation, gender and family responsibilities seem not to have a significant impact on internationalisation. Again, we should be careful in our phrasing. Gender and family responsibilities do not exert any significant impact among the PROSEPS respondents who were included in this specific set of analyses. A self-selection effect may blur the gender picture of international activities. Women academics are also more likely to be single or to have no children compared to men academics (for a recent overview of the gender barriers in European academia, see Engeli and Mügge 2020; Norris 2020b). They also remain severely underrepresented within the full professorship rank. The career stage of scholars shows some effect, in a negative direction on the possibility to travel abroad for long research and teaching stays, and in a positive direction on the other two profiles. Organisational and financial support from the academic institution of affiliation seems to matter substantively, especially for the networked researcher and for the traveller. This is a very important finding, as it is something on which academic institutions can act with relatively straightforward policies. If the internationalisation of their affiliates is believed to be a valuable asset, universities should be confident that investing resources to this aim is likely to produce visible effects.

Last but not least, geography matters too. The uneven distribution of resources between universities belonging to different European areas represents another obstacle for political scientists of southern and eastern countries to fully take part in the international debate, and even more so for countries outside the EU. The fact that scholars from the disadvantaged areas travel more than their colleagues from Northern European universities does not contradict this finding. There are certainly benefits from a circulation of political scientists for long-term visiting positions in foreign institutions. Nevertheless, problems arise for building a truly European Political Science when circulation goes one-way only (see also Norris 2020b about the implications for global political science). It is also likely that this mobility requirement creates, in turn, significant disparities within the same academic system between the scholars who get the institutional support needed for internationalisation and the ones who do not. We cannot underestimate the detrimental consequences of this unidirectional mobility towards the Northern European academic systems: the brain-drain effect that then cascades into weakening political science as a discipline and political scientists as scholars in the concerned academic systems, the personal costs to the scholars who see little alternative than leaving their home country to pursue their profession, and the significant detriment to efforts for cumulative science. As a result, we remain far from achieving a truly European Political Science.

## Footnotes

1 See https://www.cost.eu/actions/CA15207/.

2 The complete survey questionnaire, the detailed methodological information, and the main descriptive results can be accessed at http://proseps.unibo.it/action/deliverables. Ioannis Andreadis, José Real-Dato, Luca Verzichelli, and Giulia Vicentini led the web engineering of the survey and the data cleaning process. We acknowledge that our sample could be more “internationalised” than the population on European political scientists, as an effect of self-selection. However, bear in mind that we are not interested here in assessing the absolute level of internationalisation. We focus instead on detecting the existence of different types of internationalised scholars and their determinants. We are confident our sample is well suited, though certainly not perfect, to catch those differences.

3 Northern Europe includes respondents from Belgium, Denmark, Finland, Germany, Iceland, Ireland, Luxembourg, Norway, Sweden, Switzerland, The Netherlands, UK, and Austria. The Mediterranean countries are France, Greece, Italy, Spain, and Portugal. CEE countries that are members of the EU are Bulgaria, Croatia, Czech Republic, Estonia, Hungary, Latvia, Lithuania, Poland, Romania, Slovakia, and Slovenia. CEE countries that are not members of the EU are Albania, Bosnia-Herzegovina, Macedonia, Moldova, Russia, and Serbia.

4 There may be other family members who need care from the respondents, such as elderly relatives for example. This information is not available in the PROSEPS survey.
